# Case of Gunshot Wound, with Remarks

**Published:** 1830-08

**Authors:** J.W. Heustis

**Affiliations:** Cahawba, Alabama.


### GUNSHOT WOUND.

*Case of Gunshot Wound, with Remarks.* By J. W. Heustis, M.D. of Cahawba, Alabama.*

On the 16th of January, 1824, L. Roberts, of this place, was shot by the discharge of a small cannon, on board one of the steamboats, used as a signal for arrival and departure. He was standing with others on the bank of the river, and the piece being pointed incautiously to the shore, wounded by its discharge the subject of this article in the back. It was supposed that the wadding of the gun was made of some old clothes having buttons on them, from the circumstance and appearance of the wound, which was on the right of the spine, just below the crest of the ileum, about the size of a quarter of a dollar. Upon examination, it appeared that the intestinal canal was broken, as the contents of the bowels were discharged freely at the wound. The place was dressed, and quietude and a recumbent posture were enjoined. Having a patient some miles distant, I did not see Roberts again until the next day. On my return, I was told that a consultation of physicians had been held upon his case, which was considered desperate, and that, with the approbation of the patient, they had come to the conclusion of opening the abdomen, searching for, taking up, and sewing together the wounded portion of intestine. The physicians who held this consultation were gentlemen highly respected in their profession and by society at large. As they made known to me the result of their deliberations, I begged leave to differ from them in opinion, as the practice proposed was unauthorised by example, and at variance with the advice of the best surgical writers on the subject. Had the injured extremities or portion of intestine protruded from the wound, there could then have been no objection to uniting by suture the lacerated bowel, having previously pared off the jagged and deadened circumference, and then securing it to the external orifice. But here we should have been operating completely in the dark; a considerable thickness of adipose and muscular substances would have been required to be cut through before the intestine could be arrived at; besides, the bowels might have been wounded in more places than one, and the situation of these wounds was therefore a matter of uncertainty? The search might have been tedious and unsuccessful; or if successful, and the injured part had been properly united and secured, was there not reason to apprehend that such an extensive wound, the exposure of the abdominal

* American Journal of Med. Sciences.

cavity, and rough handling, would bring on dangerous or fatal inflammation of the peritoneum and intestines? I gave it as my opinion, that, however small it might be, the patient's only chance consisted in avoiding an operation so dangerous and uncertain, and, with proper medical treatment and external dressings, leaving the event to the curative operation of nature; to that *vis medicatrix,* so surprisingly presiding over human health and existence, from the first quickening of animal life to its final extinction. My advice was taken. The patient was laid upon a mattress, on his back, partially inclined to the injured side; and as the contents of the bowels escaped by the wound, a corresponding hole was cut through the bedding, and as much cleanliness preserved as the nature of the case would admit. He was now regularly attended and dressed by Mr. HOGAN, a student of Dr. CASEY's, and myself, every day. No alarming general symptoms ensued; though for nearly three weeks substances taken by the mouth and enemata were discharged in greater or less quantity by the wound. At length the residuary mass began to resume its natural channel, the external wound gradually healed and contracted, and a portion of the crest of the ileum, of the size of the finger presented and was contracted from the external opening. Finally, the wound in the course of four or five months healed up entirely, and the man has since enjoyed very sound and comfortable health.

It is scarcely necessary to say, that in the treatment of this case nothing but gentle laxatives, enemata, and the mildest articles of diet, as soup and gruel, were allowed. The patient was an old soldier, a man of much firmness and resolution, and upon whom calamity, or the prospect of death, appeared to make little or no impression.

Against the practice of opening the patient and searching for the wounded intestine, Mr. JOHN BELL makes the following judicious observations. "When there is a wounded intestine, which we are warned of only by the passing of the feces, we must not pretend to search for it, nor put in our finger, nor expect to sew it to the wound, but we may trust that the universal pressure which prevents the great effusion of blood and collects the blood in one place, that very pressure which always causes the wounded bowel and no other to protrude, will make the wounds, the outward wound and the inward wound of the intestine, oppose each other, point to point, and if all be kept there quiet, though but for one day, so lively is the tendency to inflame, that the adhesion will be begun which is to save the patient's life."

A case similar to the one above is given in the New York Medical Journal for Dec. 1828, p. 574, from the Memoirs of the American Academy of Arts and Sciences; and similar instances are recorded by a variety of authors.

It might be supposed that wounds penetrating the intestines through the parietes of the abdomen, would prove generally and necessarily fatal, yet even against this nature has in a great measure guarded, by leaving no vacuum or unoccupied space within the general peritoneal cavity, and by the uniform and equable pressure and support which the diaphragm, the abdominal parietes, and the contained viscera afford each other. When the stomach and bowels are in a state of repletion, they are applied in close contact to the peritoneal envelope, and, when the former are empty, this contact and adaptation are preserved by the contraction and elasticity of the latter: besides each convolution and portion of the intestinal canal has its appropriate location, so that, in case of injury, the external wound corresponds with the internal.

Another way in which nature consults her own safety and preservation, is by the process of adhesive inflammation. One of the chief and the essential means of reunion, in cases of solution of continuity in the animal body, is the deposition of lymph, which becoming organized by the formation of new vessels and nerves, unites and envelopes contiguous surfaces and parts; so that in case the wounded portion of intestine, by rest and position, can preserve its natural adaptation to the external opening for a few hours, the danger and possibility of displacement is effectually guarded against and prevented. "Soldiers," says Mr. John Bell, "recover daily from the most desperate wounds; and the most likely reasons that we can assign for it, are the fulness of the abdomen, the universal, equable, and gentle pressure, and the active disposition of the peritoneum, ready to inflame with the slightest touch. The wounded intestine is, by the universal pressure, kept close to the external wound, and the peritoneum and intestine are equally inclined to adhere. In a few hours that adhesion is begun which is to save the patient's life, and the lips of the wounded intestine are glued to the lips of the external wound. Thus is the side of the intestine united to the inner surface of the abdomen, and though the gut casts out its feces while the wound is open, though it often casts them out more freely while the first inflammation lasts, yet the feces resume their regular course whenever the wound is disposed to close."

Although nature thus guards against the effusion of the contents of the bowels into the cavity of the abdomen, yet we can readily suppose that this may sometimes be prevented by the severity and extent of the wound, dividing the intestines to such a degree, or in such a situation, as to cause their contents to be immediately discharged within the general cavity: this will be the more liable to happen should the alimentary canal be in a state of repletion and distention. Numerous cases are recorded by surgical writers, in which persons have been stabbed and shot through the body without fatal consequences, and sometimes without being followed by any serious or alarming symptoms. It is very probable that, in some instances of this nature, the slippery and mobile convolutions of the bowels may elude the ball or the point of the weapon, yet in many, and perhaps the majority of cases, there can be little doubt that the intestines have been wounded to a greater or less extent, and that the discharge of matter from them into the general cavity of the abdomen is prevented by that uniform and equable pressure and support afforded by the juxtaposition and adaptation of contiguous and surrounding parts, and, as already stated, the adhesive inflammation which speedily takes place, prevents all danger of effusion after the lapse of a few hours.

I am aware that in these observations I have little claim to originality, yet, as a bare detail and history of isolated cases without suitable remarks and explanations, is comparatively uninstructive, the above considerations, I trust, will not be thought inappropriate.

